# Efficiency of mitochondrial genes and nuclear *Alu* elements in detecting human DNA in blood meals of *Anopheles stephensi* mosquitoes: a time-course study

**DOI:** 10.1186/s13071-023-05884-0

**Published:** 2023-08-14

**Authors:** Fahimeh Talebzadeh, Masoud Ghadipasha, Jaber Gharehdaghi, Reza Raoofian, Kamal Azam, Mona Koosha, Mohammad Ali Oshaghi

**Affiliations:** 1 Department of Vector Biology and Control of Diseases, School of Public Health, Tehran University of Medical Sciences, Tehran, Iran; 2 Legal Medicine Research Center, Legal Medicine Organization, Tehran, Iran; 3 Department of Epidemiology and Biostatistics, School of Public Health, Tehran University of Medical Sciences, Tehran, Iran

**Keywords:** cytB, 16S rRNA, *Alu*-repeat, Time course, Blood meal, *Anopheles stephensi*, Forensic entomology, vector capacity

## Abstract

**Background:**

The time required for PCR detection of DNA in human blood meals in vector mosquitoes may vary, depending on the molecular markers used, based on the size and copy number of the amplicons. Detailed knowledge of the blood-feeding behavior of mosquito populations in nature is an essential component for evaluating their vectorial capacity and for assessing the roles of individual vertebrates as potential hosts involved in the transmission of vector-borne diseases.

**Methods:**

Laboratory experiments were conducted to compare the time course of PCR detection of DNA in human blood meals from individual blood-fed *Anopheles stephensi* mosquitoes, using loci with different characteristics, including two mitochondrial DNA (mtDNA) genes, *cytB* (228 bp) and 16S ribosomal RNA (rRNA) (157 bp) and nuclear *Alu*-repeat elements (226 bp) at different time points after the blood meal.

**Results:**

Human DNA was detectable up to 84–120 h post-blood-feeding, depending on the length and copy number of the loci. Our results suggest that 16S rRNA and* Alu*-repeat markers can be successfully recovered from human DNA up to 5 days post-blood-meal. The 16S rDNA and* Alu*-repeat loci have a significantly (*P* = 0.008) slower decline rate than the cytB locus. Median detection periods (T50) for the amplicons were 117, 113 and 86.4 h for *Alu*-repeat, 16S rDNA and cytB, respectively, suggesting an inverse linear relationship between amplicon size/copy number and digestion time.

**Conclusion:**

This comparative study shows that the* Alu*-repeat locus is the most efficient marker for time-course identification of human DNA from blood meals in female mosquitoes. It is also a promising tool for determining the anthropophilic index (AI) or human blood index (HBI), i.e. the proportion of blood meals from humans, which is often reported as a relative measure of anthropophagy of different mosquito vectors, and hence a measure of the vector competence of mosquito species collected in the field.

**Graphical Abstract:**

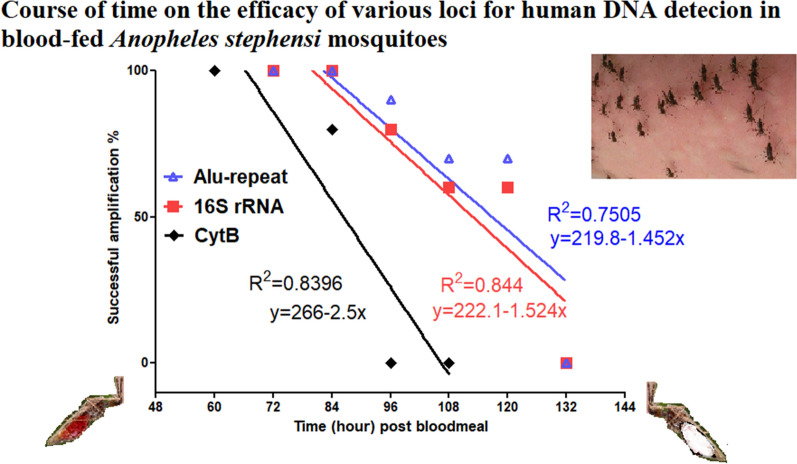

**Supplementary Information:**

The online version contains supplementary material available at 10.1186/s13071-023-05884-0.

## Background

Insect-borne diseases are major global health problems, causing hundreds of thousands of deaths per year [1], and various mosquito species are among the most important vectors of disease, being responsible for the highest transmission of human and animal parasites and pathogens. Globally widespread, mosquito-transmitted diseases include malaria, lymphatic filariasis, dengue, chikungunya, Rift Valley fever, Japanese encephalitis, West Nile virus and Usutu virus [2]. Hematophagy is present in most females of all mosquito species: they require a blood meal to produce mature eggs [3]. The extent of vector contact with an infected host or reservoir is an important factor in the transmission of mosquito-borne diseases.

Different mosquito species have wide and variable host preferences, the most important of which include humans, cattle and other mammals, but also include birds and reptiles, amphibians, worms, leeches and fish [3]. The host preference of each mosquito species shows an innate and specific pattern and may also be influenced by factors such as environmental conditions and host characteristics [2]. By detecting the source of blood in blood-feeding mosquitoes, important information can be obtained, such as the degree of contact between vector and host populations and the insect’s host preferences under natural conditions. The anthropophagic index (percentage feeding on humans) is used as component of measuring vectorial capacity for human diseases, while knowledge of other hosts can provide a measure of the relative importance of animal reservoirs of vector-borne zoonotic or enzootic infections [4]. Thus, by knowing the feeding patterns of mosquitoes on a particular host, it is possible to understand their life history, as well as the effect of host selection on their survival and reproduction and the ecology of diseases transmitted by mosquitoes. These data are also vital for operations associated with entomological surveillance and vector-monitoring, especially in the field of diseases whose etiology has an environmental component (environmental disorders) [3].

Various methods are available for determining the source of a mosquito blood meal. Previously, arthropod blood-feeding was detected by serological assays, such as the enzyme-linked immunosorbent (ELISA) method and the precipitin test [5–7]. While these assays are still used, they are insufficiently specific when attempting to identify the sources of a mosquito blood meal and often limited to determination of the order and family of the mosquitoes' host under study. New applications of PCR-based methods and the increasing amount of vertebrate DNA sequence data available in the public domain have allowed an increase in the specificity of blood-meal recognition down to the species or individual host level.

The efficacy and the success of PCR amplification are affected by several factors, including time since blood meal, PCR amplicon size, locus sequence/gene copy number, DNA extraction procedure, preservation method, storage time and blood-meal size [8–13]. Among these factors, longer time, increased amplicon size and low gene/locus copy number have inverse effects on the success of the PCR, as has been demonstrated extensively in studies designed to identify blood meals in hematophagous arthropods [14, 15].

Different DNA-based molecular markers have been used to identify ingested blood in arthropods. Mitochondrial genes (mtDNA) have been used extensively as reliable common markers to identify the source of the blood meal ingested by arthropods [16–21]. Consequently, mtDNA has been used widely in entomological, forensic and genetic studies because of their valuable features, such as lack of recombination, presence of very high copy numbers in each cell, maternal inheritance, absence of introns, existence of single-copy orthologous genes and high mutation rate [22–25]. Among the mtDNAs, cytochrome b (cytB) and 16S ribosomal RNA (rRNA) are the most applicable and are therefore appropriate for use in arthropod blood-meal identification in terms of determining the host species in phylogenetic and biodiversity studies and in identifying human individuals in forensic investigations [26–34].

Single-copy nuclear genes can also be used to identify the source of an arthropod blood meal [35]. However, when working with such a small amount of starting material, the amplification of target DNA can be more challenging with single-copy genes, thus a preferred approach is the use of *Alu* elements, which are transposable elements (TEs) that exist only in primates [36]. *Alu* repeats consist of short interspersed nuclear elements (SINEs) that replicate through LINE (long interspersed nuclear element)-mediated reverse transcription of an RNA polymerase III transcript [37]. They are the most abundant individual feature in the human genome, forming 10% of the human genome mass, with over one million copies per genome; each *Alu* element is approximately 280 bp long and always comes after a poly(A) tail of varying length. Although *Alu* repeats are not known to have a specific biological function, they have been extensively studied due to their many branches and copies in the human genome [37, 38]. There have been many reports on the use of *Alu*-based PCR amplification as a very sensitive and powerful tool for human genomic DNA identification and quantitation in forensic laboratories [39].

The objectives of this study were to compare the effects of elapsed time, copy number and amplicon size on the effective use of three different loci (mitochondrial cytB and 16S rRNA genes, and nuclear *Alu*-repeat elements) in PCR assays for tracking human DNA in blood meals of the *Anopheles stephensi* mosquito. *Anopheles stephensi* was selected for study is one of the most important malaria vectors in Asian countries and, currently, in the Horn of Africa.

## Methods

### Mosquito rearing

*Anopheles stephensi* mosquitoes were maintained at 28 °C ± 2 °C, 60 ± 10% humidity, under a 12:12-h light:dark photoperiod, and were fed only on a 10% sucrose solution before the experiments. The colony of *An. stephensi* used in this study was maintained in the insectarium at the School of Public Health, Tehran University of Medical Sciences. Eggs were hatched in about 1 l of tap water that was continuously supplemented with a few flakes of fish food until the larvae pupated. Pupae were collected and separated according to age. Adult mosquitoes aged 5–7 days were blood-fed, either artificially on membrane blood feeders or directly on male or female human volunteer hands and forearms in a cage under laboratory conditions [40].

### Sample collection

The exclusively human blood-fed mosquitoes were transferred to a cage supplied with 10% sucrose solution to reduce mortality. Individual mosquitoes were randomly chosen, anesthetized and killed by freezing at 0, 6, 12, 24, 36, 48, 60, 72, 84, 96, 108, 120 and 132 h post-feeding. Knockdown and killing times of < 10 s was achieved for each specimen. Starved male mosquitoes and ddH_2_O were used as negative controls. Specimens were stored dry in micro-tubes at − 20 °C. Prior to the experiment on PCR success rate, the head and legs were removed from each dead mosquito to reduce potential PCR inhibitors [41, 42] and to avoid high concentrations of nontarget DNA, respectively.

### DNA extraction and quantification

Genomic DNA was extracted using the protocol of the Vivantis GF-1 nucleic acid extraction kit (Vivantis Technologies, Singapore). The final elution was carried out twice in the same 20 μl of elution buffer to maximize the amount of recovered DNA. Positive controls were 0.1–3 μl human blood samples, roughly equal to the minimum and maximum amount, respectively, of a blood meal in a mosquito′s midgut [43]. These samples were taken with a lancet from a female and male donor separately and poured directly into a 1.5-ml micro-tube. Using the Vivantis GF-1 extraction kit, DNA was extracted from the male mosquito (negative controls), the human blood (positive controls) and 10 individual female mosquitoes from each post-feeding time point. DNA was quantified on a Nano-Drop 2000 spectrophotometer (Thermo Fisher Scientific, Waltham, MA, USA).

### Primers and PCR reactions

Three human-specific primer pairs (Table 1) for the cytB, 16S rRNA and *Alu*-repeat loci were used to confirm the absence of human DNA from ddH_2_O and male mosquitoes (the negative controls) and the presence of human DNA in the fed female mosquitoes and in the blood stains (positive controls). Various volumes (0.1, 1.0 or 2.0 μl) of DNA from human blood stains, as positive controls, were used to test the sensitivity and reproducibility of the three loci amplification. To test possible inhibitory substances (exocuticle and thorax) that might affect the PCR amplification, a serial dilution of human blood was performed in volumes of 0.1, 1.0, and 2.0 μl, plus a male mosquito in each sample.

**Table 1:** List of human-specific primers and thermal cycles used in the study

Loci	Primer sequence 5'-3'	Size of PCR (bp)	Modified thermal cycles	References
*Alu*-repeats	F: CGAGGCGGGTGGATCATGAGGT R: TCTGTCGCCCAGGCCGGACT	226	94 °C for 5 min; followed by 35 cycles of 94 °C for 25 s, 64.4 °C for 25 s and 72 °C for 20 s; with a final extension at 72 °C for 5 min	[39]
Cytb	F: TTCGGCGCATGAGCTGGAGTCC R: TATGCGGGGAAACGCCATATCG	228	94 °C for 2 min; followed by 35 cycles of 94 °C for 30 s, 64 °C for 40 s and 72 °C for:30 s; with a final extension at 72 °C for 8 min	[45]
16S rRNA	F: CAATTGGACCAATCTATCACC R: GTGAGGGTAATAATGACTTGT	157	94 °C for 5 min; followed by 35 cycles at 95 °C for 25 s, 65.8 °C for 25 s and 72 °C for 20 s; with a final extension at 72 °C for 5 min	[46]

*F* Forward, *R* reverse

PCR amplifications for all genes or loci were performed according to the protocol for *Taq* DNA Polymerase Master Mix (2×) (Ampliqon A/S, Odense M, Denmark). The PCR reaction was carried out in a 25-μl reaction volume consisting of 12.5 µl of Master Mix, 1 µl of each primer and 10 μl of template or ddH_2_O. The Master Mix is a premixed solution containing HotStarTaq DNA polymerase, PCR buffer and deoxynucleotide triphosphates (dNTPs), with a final concentration of 2 mM MgCl_2_ and 200 mM of each dNTP. Details of the thermal cycling of each protocol are listed in Table 1. All amplicons were visualized under ultraviolet light using a UV-transilluminator after electrophoresis at 85 V in a 3% agarose gel and staining with Safe stain. Negative and positive controls were included in each PCR.

The success rates of the PCR assay for each locus were subjected to regression analysis, and the median detection period (T_50_) [44] was designed for each amplicon using the regression equation* y* = m*x* + c, where* x* = time and m and c are constants. T_50_ is defined as the point at which 50% of amplification is expected to be successful, as predicted by the regression line. In this experiment we calculated regression lines only for critical time points, which included the last two time points with 100% success amplification, followed by the time points with < 100% success up to the lack of amplicons (0%). Regression lines were subsequently subjected to comparison by analysis of covariance (ANCOVA; general linear model [GLM]) using SPSS version 26 software package (SPSS IBM Corp., Armonk, NY, USA) to compare the rate of waning of the slopes of the three amplicons. To control the experiment, a subset of PCR products from each locus was randomly sequenced by the same forward and reverse primers used for the PCR and their identity was then determined by BLASTn searches against the GenBank nucleic acid sequence database (http://www.ncbi.nlm.nih.gov/BLAST/).

## Results

The sensitivity and reproducibility tests of the PCR for the three loci showed that all of the DNA samples (0.1, 1.0 and 2.0 µl) were positive for the expected bands of 226, 228 and 157 bp for the *Alu*-repeat, cytB and 16S rRNA, respectively, validating the DNA extraction process and showing the sensitivity and reproducibility of the amplification of the three loci using the minimum and maximum blood volumes (0.1 and 2.0 µl, respectively; Additional file 1: Fig. S1). No PCR product was obtained with the negative controls. A test was performed for the presence of potential PCR inhibitors in the mosquito DNA and for the frequency of false-negative PCR reactions in DNA from blood-fed mosquitoes. All the samples (a mix of DNA from human blood and a decapitated and amputated male mosquito) produced bands of the expected sizes corresponding to the three loci, confirming that any possible PCR inhibitors had been removed (Additional file 2: Table S1). In addition, BLASTn searches against the GenBank nucleic acid sequence database showed that the blood meals identified from mosquitoes were identical (> 99%) to human sequences.

**Fig. 1:**
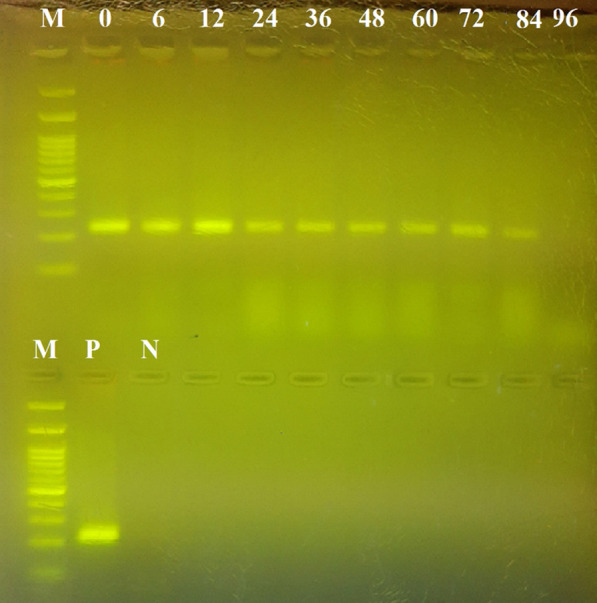
Effect of the elapsed time post-feeding on the success of PCR amplification of cytB (228 bp) from human blood meal in *Anopheles stephensi*. The numbers above the well are the elapsed time intervals (h) post-feeding. Amplification was successful up to 84 h post-feeding. CytB, Cytochrome b; M, 100-bp marker; N, Negative control; P, positive control

**Fig. 2:**
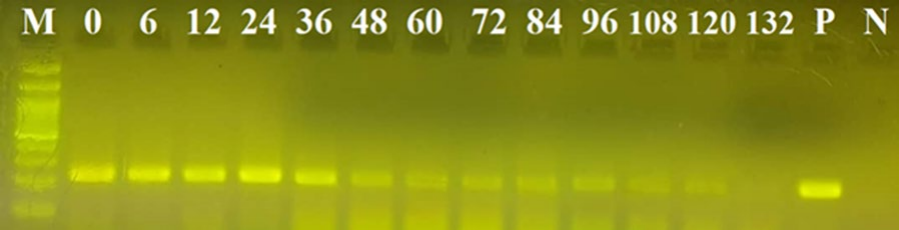
Effect of the elapsed time post-feeding on the success of PCR amplification of the *Alu*-repeat (226 bp) from human blood meal in *Anopheles stephensi*. The numbers above the well are the elapsed time intervals (h) post-feeding. Amplification was successful up to 120 h post-feeding. M, 100-bp marker; N, negative control; P, positive control

**Fig. 3:**
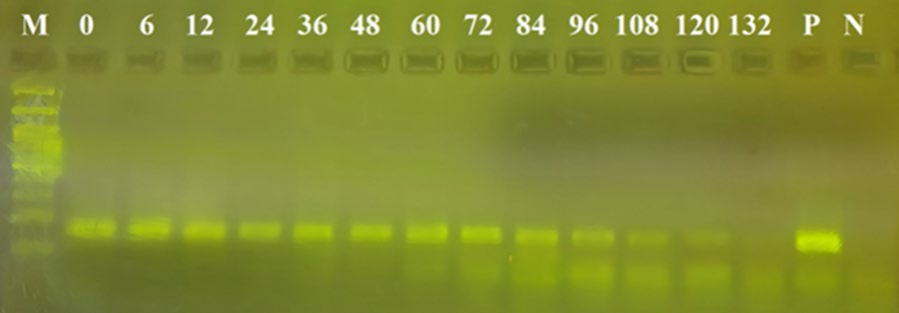
Effect of the elapsed time post-feeding on the success of PCR amplification of 16S rRNA (157 bp) from human blood meal in* Anopheles stephensi*. The numbers above the well are the elapsed time intervals (h) post-feeding. Amplification was successful up to 120 h post-feeding.M, 100-bp marker; N, negative control; P, positive control; rRNA, ribosomal RNA

The results of this experiment showed that the success rate of the amplification of human DNA decreased with increasing elapsed time post-feeding. Specifically, human DNA could be traced in the blood meals of *An. stephensi* using the cytB gene for up to 84 h post-feeding; the 16S rRNA locus could be used up to 120 h post-feeding; and the *Alu*-repeats in human DNA could be detected up to 120 h post-feeding (Figs. 1, 2, 3). The maximum period of detection was similar (120 h) for the shortest amplicon (16S rRNA, 157 bp) and the amplicon with the highest copy number (*Alu*-repeat, 226 bp).

**Fig. 4:**
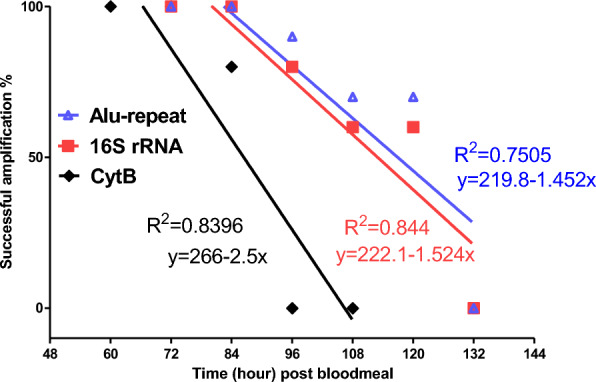
Regression lines for time course and successful amplification of human DNA within the midgut of *Anopheles stephensi* for three human-specific molecular markers (mtDNA cytB and 16S rRNA, and nuclear *Alu*-repeat) with 228-, 157- and 226-bp amplicons, respectively. CytB, Cytochrome b; rRNA, ribosomal RNA

The success of PCR amplification was 100% for up to 84-, 84- and 72-h post-feeding for 16S rRNA, the *Alu*-repeat and the cytB gene, respectively. The time points at which 50% of the PCR amplification is expected to be successful (T_50_), as calculated by the regression lines for the critical time points, were 117 h for the *Alu*-repeat (226 bp), 113 h for the 16S rRNA (157 bp) and 86.4 h for the cytB (228 bp) amplicons (Fig. 4). A comparison between the regressions for the two mitochondrial amplicons (cytB and 16S rRNA), which are assumed to have the same copy number in the cells, showed the PCR success rate of the smaller amplicon (16S rRNA) to be significantly higher (GLM, *P* = 0.008, *F* = 11.497, *R*^2^ = 82.5%). A comparison between the regressions for the cytB and *Alu*-repeat amplicons showed that the PCR success rate of the higher copy-number amplicon (*Alu*-repeat) was also significantly higher than that for the cytB amplicon (GLM, *P* = 0.008, *F* = 11.687, *R*^2^ = 79.5%). However, the comparison between the regressions for the 16S rRNA and *Alu*-repeat amplicons showed that the PCR success rate of the higher copy-number amplicon (*Alu*-repeat) was only slightly higher than that of the 16S rRNA amplicon, with the difference lacking statistical significance (GLM, *P* = 0.638, *F* = 0.236, *R*^2^ = 79.7%). The logistical regression found that the probability of obtaining successful amplification for cytB, 16S rRNA and *Alu*-repeat loci was, respectively, 30%, 18.3% and 17.4% less for every 12-h increase in post-feeding interval at their critical time points.

## Discussion

Here we propose a method for identifying human DNA in the blood meal of *An. stephensi* mosquitoes that involves amplification of the human-specific regions of cytB, 16S rRNA and *Alu* repeats, without the need to sequence the PCR products. We show that the success of the amplification over time is mainly dependent on amplicon size and locus copy number, with smaller amplicons (16S rRNA) and higher copy number loci (*Alu*-repeats) being detectable for longer periods of time after blood-feeding.

The PCR amplification of either mitochondrial 16S rRNA and or nuclear *Alu*-repeats in this study proved to be very efficient for identifying the origin of mosquito blood meals. We were able to trace human DNA in *An. stephensi* mosquito blood meals up to 120 h post-feeding. The use of this mitochondrial marker thus has the potential to be extremely useful for use in species determination and in individual human identification, for epidemiological entomology studies and also for forensic purposes [25, 46, 47]. In addition, the *Alu* repeats are a naturally amplified source of human genetic information and robust tools for sensitive human DNA detection and quantitation, as has previously been demonstrated in a forensic study [39].

In the present study, we demonstrated that human DNA can potentially be amplified by PCR, even when it has been degraded by digestion in the mosquito gut, because human DNA was detectable in *An. stephensi* for up to 84–120 h post-feeding, depending upon the locus used. The stage of blood digestion progressed from the fully fed period (fresh) to the semi-gravid period and then to fully gravid period within the first 2–3 days (48–72 h) post-feeding under the laboratory conditions (28 °C ± 2 °C), and visual validation of blood in the mosquito abdomen was also possible during the fresh and semi-gravid periods. However, our results show that the source of the blood meal could be identified from DNA, even when the blood-meal residue in the abdomen was no longer distinguishable by eye. This result is consistent with the findings reported by Replogle et al*.* [48], who showed that it was possible to successfully genotype individual human blood donors using mtDNA obtained from excrement of the pubic louse (*Pthirus pubis* L.) (Phthiraptera, Pthiridae), even when no DNA from the blood meals remained in the gut, thus demonstrating the potential of the method for the amplification of DNA degraded by digestion.

We showed here that short amplicons with high copy number can be recovered from a mosquito blood meal up to 120 h (5 days) post-feeding. This is consistent with a short amplicon (103–118 bp) assay for the identification of sand fly blood meal, which could detect various small amounts of the host DNA up to 120 h after blood-feeding [49]. However, the use of longer amplicons generally permits detection of host DNA for 1–4 days post-feeding [8, 35, 50–54]. In forensic studies, short amplicons of short tandem repeat (STR) alleles (normally < 200 bp) are used to identify an individual from blood or tissue isolated from insects [37, 38]. However, the use of short STR amplicons has only been shown to allow DNA recovery for 15–88 h post-blood-meal [50, 55]. Our data suggests that exploring the use of shorter STR alleles with higher copy numbers could increase the recovery time of human DNA from mosquitoes for criminal investigation.

The results of this study demonstrate that longer amplicons are more subject to degradation than short amplicons (Fig. 4). In the *An. stephensi* blood meal, the DNA degradation rate of the short 16S rRNA amplicon is comparable with that of the longer, high copy number *Alu*-repeat, suggesting that the high copy number of the *Alu*-repeat compensates for its longer amplicon size.

In addition to the time post-blood meal, the locus copy number and the amplicon size, other factors may affect the success of blood-meal source identification using PCR. These include the PCR-inhibitory substances present in insect tissue, especially those in the cuticle, head and thorax [56, 57], as well as those in blood, such as heme [5]. In this study we used a combination of male mosquitoes and human blood to test the inhibitory effects of these materials on our PCR reactions; it is evident from the absence of false negatives that the PCR reactions were not hindered by those materials present in the insects or the blood. It appears that across the time course of the experiment, the combined activities of digestive enzymes and nucleases play the main roles in degrading the ingested human blood, so that human DNA could not be obtained after 5 days post-blood meal.

## Conclusion

We describe here an assay with greatly enhanced sensitivity and specificity for identifying the source of the blood meals of hematophagous mosquitoes, which allows detection of human DNA in a single round of PCR. We demonstrate for the first time that the use of 16S rRNA and/or *Alu*-repeat elements permits the identification of human blood sources from *An. stephensi* mosquitoes up to 5 days post-blood meal. It is evident that selecting short amplicons and high copy number loci of the human host will increase the longer-term success rate of PCR. However, since DNA degradation over time is a concern, we recommend exploring combinations of high copy number loci and shorter amplicons to maximize the recovery of human DNA from hematophagous insects for longer times post-blood meal.

### Supplementary Information

**Additional file 1: Figure S1.** The sensitivity and reproducibility tests of the PCR for the three loci with DNA samples 2 μl (No. 1 above the wells) and 0.1 μl (No. 2 above the wells), No 3 above the wells is negative control (ddH_2_0). Panel A: cytB (228 bp), panel B: Alu-repeat (226 bp), and panel C: 16S rRNA (157 bp).

**Additional file 2: Table S1.** Results of PCR amplification of Alu-repeat, 16S rRNA and CytB loci for validating probable PCR inhibitors in the human blood and mosquito. HB, Human blood; DC-AP, decapitated and amputated; Mos, mosquito.

## Data Availability

The datasets supporting the findings of this article are included within the article.
