# Supplementary figures and images for: Efficiency of mitochondrial genes and nuclear Alu elements in detecting human DNA in blood meals of Anopheles stephensi mosquitoes: a time-course study
Source: Parasit Vectors. 2023 Aug 14;16:284. doi: 10.1186/s13071-023-05884-0 (PMC10426119; doi:10.1186/s13071-023-05884-0)

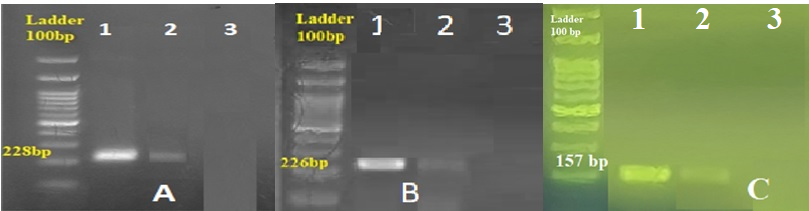

Supplement: Supplementary file 1 — Additional file 1: Figure S1. The sensitivity and reproducibility tests of the PCR for the three loci with DNA samples 2 μl (No. 1 above the wells) and 0.1 μl (No. 2 above the wells), No 3 above the wells is negative control (ddH20). Panel A: cytB (228 bp), panel B: Alu-repeat (226 bp), and panel C: 16S rRNA (157 bp). [file 13071_2023_5884_MOESM1_ESM.tif]
