# Supplementary material for: Efficiency of mitochondrial genes and nuclear Alu elements in detecting human DNA in blood meals of Anopheles stephensi mosquitoes: a time-course study
Source: Parasit Vectors. 2023 Aug 14;16:284. doi: 10.1186/s13071-023-05884-0 (PMC10426119; doi:10.1186/s13071-023-05884-0)
Supplement: Supplementary file 2 — Additional file 2: Table S1. Results of PCR amplification of Alu-repeat, 16S rRNA and CytB loci for validating probable PCR inhibitors in the human blood and mosquito. HB, Human blood; DC-AP, decapitated and amputated; Mos, mosquito. [file 13071_2023_5884_MOESM2_ESM.docx]

**Table S1**: Results of PCR amplification of Alu-Repeat, 16Sr RNA, and CytB loci for validating probable PCR inhibitors in the human blood and mosquito. HB: human blood, DC-AP: decapitated and amputated, Mos.: mosquito).

| Locus | No of DNA template tested | | | False negative | False positive |
| --- | --- | --- | --- | --- | --- |
|  | HB plus DC-AP male Mos. | HB (positive  control) | Male Mos. (negative  control) |  |  |
| Alu-Repeat | 10 | 10 | 10 | 0 | 0 |
| 16Sr RNA | 10 | 10 | 10 | 0 | 0 |
| CytB | 10 | 10 | 10 | 0 | 0 |
